# KTK MOTOR TEST: REVIEW OF THE MAIN INFLUENCING VARIABLES

**DOI:** 10.1590/1984-0462/;2019;37;3;00013

**Published:** 2019-06-19

**Authors:** Whendel Mesquita do Nascimento, Nayana Ribeiro Henrique, Marcelo da Silva Marques

**Affiliations:** a Department of Physical Education and Physiotherapy, Universidade Federal do Amazonas, Manaus, AM, Brazil.; b Laboratory of Sports Psychosociology, School of Physical Education and Sports, Universidade de São Paulo, São Paulo, SP, Brazil.; c Laboratory of Energy Determinants and Sports Performance, School of Physical Education and Sports, Universidade de São Paulo, São Paulo, SP, Brazil.

**Keywords:** Motor activity, Motor assessment, Child, Atividade motora, Avaliação motora, Criança

## Abstract

**Objective:**

To analyze the scientific literature regarding the effects of external variables on KTK motor test scores and to verify which motor tests are associated with KTK.

**Data sources:**

Four databases (PubMed, Science Direct, Scientific Electronic Library Online - SciELO - and Latin American and Caribbean Health Sciences Literature - LILACS) were used to search for studies in which the descriptors *Körperkoordinationstest für Kinder* and *KTK* were presented in the title, abstract and keywords. Inclusion criteria were: articles published in English or Portuguese from January 2006 to December 2016; free access to the article in full and texts available online; presenting the descriptor terms mentioned above in the title, abstract or keywords; containing sample with children and adolescents aged 4 to 16 years old; being indexed in a journal with a rating of B2 or higher (WebQualis; Qualis 2016) for the area of physical education. The following were excluded: studies in books, chapters of books, theses and dissertations; duplicate scientific articles; conference summaries; articles published in proceedings and abstracts of congresses.

**Data synthesis:**

After the three stages of selection (identification, screening and eligibility) and the criteria proposed at the PICOS scale, 29 studies were included in this review.

**Conclusions:**

Body composition and the regular practice of physical activities were the variables that presented the greatest influence on KTK. It is important that health professionals working with the pediatric public encourage regular physical activity to improve body composition and, thus, to obtain better KTK scores. Additionally, the Movement Assessment Battery for Children (M-ABC) test had the highest positive correlation with the KTK test.

## RESUMO

**Objetivo:**

Analisar a literatura científica quanto aos efeitos de variáveis externas nos escores do teste motor KTK e verificar quais testes motores se associam ao KTK.

**Fonte de dados:**

Quatro bases de dados (PubMed, Science Direct, Scientific Electronic Library Online - SciELO - e Literatura Latino-Americana e do Caribe em Ciências da Saúde - LILACS) foram utilizadas para busca de estudos em que os descritores *Körperkoordinationstest für Kinder* e KTK foram apresentados no título, no resumo e nas palavras-chave. Os critérios de inclusão foram: artigos publicados em língua inglesa ou portuguesa de janeiro de 2006 a dezembro de 2016; acesso livre na íntegra e textos disponíveis *on-line*; apresentação dos termos descritores supracitados no título, no resumo ou nas palavras-chave; conter amostra com crianças e adolescentes de quatro a 16 anos; estar indexado em uma revista com classificação igual ou superior a B2 (WebQualis; Qualis 2016) para a área de educação física. Foram excluídos: estudos em livros, capítulos de livros, teses e dissertações; artigos científicos duplicados; resumos de conferências; artigos publicados em anais; e resumos de congressos.

**Síntese dos dados:**

Após três etapas de seleção (identificação, triagem e elegibilidade) e os critérios propostos na escala PICOS, 29 estudos foram incluídos nesta revisão.

**Conclusões:**

A composição corporal e a prática regular de atividades físicas foram as variáveis que apresentaram maior influência no KTK. Parece ser importante que os profissionais da saúde que atuam com o público pediátrico incentivem a prática regular de atividades físicas para melhorias da composição corporal e, assim, para a obtenção de melhores escores no KTK. Adicionalmente, o teste Movement Assessment Battery for Children (M-ABC) apresentou a maior correlação positiva com o teste KTK.

## INTRODUCTION

The scientific literature reports several tests for the analysis of the motor performance pattern in children. However, some variables may influence the results of certain tasks that are part of these tests, such as, for example, body composition in the *Körperkoordinationstest für Kinder* (KTK) test battery.^1^ Considering that the objective of motor tests is providing results with greater reliability of the main findings^2^ ^,^ ^3^ and that the coordinative development of children has the characteristic of predicting physical activities in the later stages of life,^4^ there is a need for conducting scientific analyzes that seek to verify the influence of intervening variables (for example, physical activity level, body composition) in these tests, as well as to help in the understanding of an analysis method with greater reproducibility.

Investigations conducted with the application of motor tests in children seek to assist in the early diagnosis of motor disorders. This fact is justified by the relevance of a greater possibility of effective interventions as soon as movement difficulty is identified, aiming both at the decrease of these disorders^3^ ^,^ ^5^ ^,^ ^6^ ^,^ ^7^ due to the evident neuroplasticity and the relearning of the correct movement pattern.^8^ ^,^ ^9^ ^,^ ^10^

In addition to mechanical limitations, motor disorders also seem to influence the interpersonal relationships of children. The literature suggests that, during recreational sports, children with motor disorders tend to be excluded or even self-excluded from such practices,^3^ influencing involvement in motor practices such as games and sports in later life.^11^ In addition, Lopes et al.^12^ infer that children with less developed coordination, according to KTK data, are at increased risk of being overweight or obese adults, mainly due to practicing less physical activities when compared to those with more developed coordination.

In fact, different variables may influence the results of the KTK motor test. Previous studies using this test have verified that factors such as the maturational phase,^13^ the environment where the child is inserted^14^ and the body composition,^15^ the latter even in active children,^16^ influence positively or negatively the motor coefficient or the task scores. In this sense, it is reasonable to infer that innumerable extrinsic factors may influence the results of a motor test such as KTK, but it is still unclear in the literature which factors exert more influence on KTK scores.

Initially proposed in 1974 by Kiphard and Schilling,^17^ the KTK’s main objective is to diagnose children with movement difficulties, including motor coordination components such as balance, rhythm, strength, laterality and agility,^3^ ^,^ ^17^ with an approximate duration of 20 minutes. The test consists of four tasks:

- Task 1: balance beam.
- Task 2: single-lever jumps.
- Task 3: lateral jumps.
- Task 4: transfers on platforms.^18^

It is suitable for children with a typical motor development pattern, as well as for children with brain disorders, behavioral problems, or learning difficulties.^18^ Thus, KTK gained prominence given its ease of application and reading of results, making it one of the most commonly applied tests to assess motor coordination^16^ not only by teachers, but also by other health professionals who work with the pediatric population.^19^

In addition to KTK, other tests are proposed in the literature to access motor performance in children. The Movement Assessment Battery for Children (M-ABC), the Test of Gross Motor Development 2 (TGMD-2) and Motor-Proficiency-Test for Children between 4 and 6 Years of Age (MOT 4-6) are the most common. The M-ABC assesses the level of development of daily life movement skills (manual dexterity, ball skills and balance), focusing detecting delays or deficits in the development of these skills in children. It also measures the level of treatment evolution and has a duration of 20 to 30 minutes.^19^ ^,^ ^20^ ^,^ ^21^ The TGMD-2 measures the gross movement performance based on movement skills. It is used to identify children who are significantly behind their peers in gross motor performance, to plan programs to improve skills in children who present such delays and to evaluate changes as a function of age increase, experience, education or intervention by the health professional. It lasts between 15 and 20 minutes.^19^ ^,^ ^22^ Finally, the MOT 4-6 was developed to contribute to the early detection of deficiency in fundamental (fine and gross) movement skills as it is only used in children aged four to six. The test is rooted in KTK’s principle, for which adaptations were made in order to make the test appropriate for the specific age range of pre-school children. It lasts between 15 and 20 minutes.^19^ ^,^ ^23^

Considering the importance of the reproducibility of the findings in the different tests, some authors conducted comparisons among the different tests^24^ ^,^ ^25^ and reported inconsistencies mainly due to the greater or lesser influence of external variables on the different tasks proposed in the tests. Thus, given KTK’s wide applicability and good acceptance in the scientific community, it is important to also verify which other tests have an association with KTK, seeking to establish more valid and reproducible data, allowing greater comparability between the studies.

Given this and considering the complexity of the constructs related to the motor behavior of children, this review aims to identify the variables that exert significant influence on the KTK motor test and, secondarily, to review which other tests reported in the literature have more similarities or differences when associated to KTK.

## METHOD

Aiming at the objectives of this study, the methodology adopted was a systematic review of the scientific literature. The process of identifying pre-selected and selected studies was carried out independently by two researchers, aiming to guarantee scientific rigor.

For the selection of articles, a retrospective search of manuscripts published from January 2006 to December 2016 was conducted in PubMed (US National Library of Medicine, National Institutes of Health), Science Direct (Elsevier Group), Latin American and Caribbean Health Sciences Literature (LILACS) and Scientific Electronic Library Online (SciELO). The terms adopted, included in the list of Health Sciences Descriptors (DeCs) and Medical Subject Headings (MeSH), were *Körperkoordinationstest für Kinder* and KTK, selected in the title, abstract or keywords.

The following inclusion criteria were adopted: only articles published in English or Portuguese; free access in full and texts available online; having the descriptor terms mentioned above in the title, abstract or keywords; including children and young people aged four to 16 years as participants in the study; being indexed in a journal with a rating of B2 or higher, according to the WebQualis (Qualis 2016) evaluation for the area of physical education. On the other hand, were excluded: studies in books, chapters of books, theses and dissertations; duplicate scientific articles; conference summaries; articles published in proceedings and abstracts of congresses. Initially, the titles related to the theme were shows. The studies were then selected by reading the titles, based on previously established inclusion and exclusion criteria. This was the first stage in the selection process.

**Figure 1:**
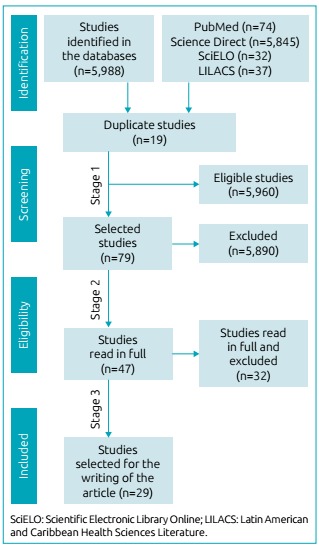
Flowchart of the selection process of scientific articles in the revision, adapted from Preferred Reporting Items for Systematic Reviews and Meta-Analyses.

The strategy of searching the studies included in this review (Figure 1) was conducted according to the proposal presented by the Preferred Reporting Items for Systematic Reviews and Meta-Analyzes (PRISMA) in 2009^26^ and according to the eligibility criteria of PICOS^26^ ((participants, intervention, comparison, outcomes and study design). Thus, PICOS criteria were (Table 1): children and young *participants* (age range between 4 and 16 years). The *intervention* considered was the application of KTK and/or the M-ABC, TGMD-2 and MOT 4-6 tests. Regarding the *comparison*, it was observed whether or not there was a comparison between the effects of different external variables or whether there was a comparison between the motor tests. Regarding the *results*, the external factors that influenced the KTK scores were analyzed. When considering the *study design*, intervention and observational studies were considered. These strategies have already been widely adopted and recommended for systematic reviews.^27^ ^,^ ^28^ Finally, studies that fulfilled the mentioned criteria but used individuals with some kind of physical or mental disability were excluded from this review.

**Table 1:** Participants, intervention, comparison, results, design.

Component	Details
Participants	Human (children and teenagers, aged 4 to 16 years)
Intervention	Application of the KTK and/or M-ABC, TGMD-2 and MOT 4-6 tests
Comparison	Effects of external variables on the KTK test or comparisons with other tests
Results	External factors that influenced the KTK; association or not when compared the KTK to other tests
Design	Intervention or observational

KTK: *Körperkoordinationstest für Kinder*; M-ABC: Movement Assessment Battery for Children; TGMD-2: Test of Gross Motor Development 2; MOT 4-6: Motor-Proficiency-Test for Children between 4 and 6 Years of Age.

Once this was done, a detailed reading of the abstracts was carried out, and then the articles whose summaries did not meet the mentioned eligibility criteria were excluded. This was considered the second stage of selection. Finally, in the third stage of the process, the remaining texts were evaluated in their entirety. In addition, the list of references of the selected ones was analyzed in order to look for other relevant manuscripts.

For the moment, it is important to note that, when the results of the studies reported effects, it was analyzed whether there was a negative or positive effect. A positive effect is understood as an improvement on the KTK scores that the authors reported to be related to the external variable investigated. On the other hand, a negative effect is understood as worse KTK scores that the authors reported to be related to the external variable investigated.

## RESULTS

At the end of the first stage of searches in the databases, 5,988 papers were found: 74 in PubMed, 5,845 in Science Direct, 32 in SciELO and 37 in LILACS. Next, the articles were filtered based on the criteria proposed in the second selection stage, and thus, 79 articles were selected (25 in PubMed, 20 in Science Direct, 16 in SciELO and 18 in LILACS). After conducting the analyses in the third stage (full reading of the 79 articles), 50 articles were excluded because they did not fully meet all criteria and filters proposed for the construction of this study. Thus, 29 articles were selected to compose the final version of this systematic review (Figure 1).

Tables 2, 3 and 4 summarize the included studies based on the three stages presented. It noteworthy that more than half of the studies selected (20 manuscripts) have been published in the last five years, showing a possible scientific interest in the subject, keeping it current.

**Table 2:** Studies that evaluated the influence of external factors on the scores of the *Körperkoordinationstest für Kinder*.

1^st^ Author	Sample	Methodological characteristics	Main findings
Antunes et al.^1^	619 ♂ and 657 ♀ (6-14 years)	Analysis of GMC in eutrophic, overweight and obese children	Eutrophic children had better scores than their overweight and obese peers
Chagas et al.^31^	21♂ and 35♀ (12-14 years)	GMC and BMI analysis in eutrophic, overweight and obese children, controlled by PA levels	Eutrophic children had better scores than their overweight and obese peers regardless of PA levels
Chaves et al.^44^	128 ♂/♀ (5-14 years)	Analysis of the relations between environmental factors and MC	Environmental factors directly influenced the KTK performance
D’Hondt et al.^15^	454 ♂ and 500 ♀ (5-12 years)	GMC analysis in eutrophic, overweight and obese children	Eutrophic children had better scores than their overweight and obese peers
D’hondt et al.^33^	48 ♂ and 24 ♀ (7-13 years)	Analysis of the evolution of GMC in overweight and obese children after a weight reduction program	Children who lost more weight developed significantly in the KTK scores when compared to pre-intervention scores
D’hondt et al.^50^	100 ♂/♀ (6-10 years)	Longitudinal analysis of GMC in eutrophic, overweight and obese children	Eutrophic children obtained better scores according to maturation when compared to their overweight and obese peers
D’hondt et al.^40^	383 ♂ and 371 ♀ (7-13 years)	Longitudinal analysis of GMC in children of different levels of BC	Higher BC negatively influenced GMC
Debrabant et al.^48^	40 ♂ and 40 ♀ (5-12 years)	Speed of anticipatory motor response and motor performance in the KTK jump task	The increase of the anticipatory response according to age correlates with motor performance in jumping
Deus et al.^32^	143 ♂ and 142 ♀ (6-10 years)	To analyze the effects of the environment and the levels of PA on MC	Children with higher levels of physical activity presented better motor performance
Freitas et al.^35^	213 ♂ and 216 ♀ (7-10 years)	Analysis of the relationships between bone maturation and GMC	Bone maturation has insignificant influence on GMC
Giagazoglou et al.^8^	104 ♂ and 96 ♀ (8-9 years)	Analysis of the effects of a physical exercise program on the GMC of children with motor disorders	The intervention proved to be efficient to promote functional improvements in children with motor disorders

♂: boys; ♀: girls; GMC: gross motor coordination; BMI: body mass index; PA: physical activity; MC: motor coordination; BC: body composition; KTK: *Körperkoordinationstest für Kinder*.

**Table 3:** Studies (n=23) that assessed the influence of external factors on the scores of the *Körperkoordinationstest für Kinder.*

1^st^ Author	Sample	Methodological characteristics	Main findings
Hanewinkel et al.^25^	22 ♂ and 19 ♀ (5-12 years)	Analysis of the KTK sensitivity for motor disorders	The KTK has been shown to be sensitive and a valid tool for detecting motor disorders in children with joint hypermobility
Laukkanen et al.^9^	91 children (4-7 years)	Longitudinal analysis of the relationships between physical activity level and KTK performance	Children stimulated by the family to engage in various physical activities scored higher on the KTK at the end of a year
Laukkanen et al.^42^	38 ♂ and 46 ♀ (5-8 years)	Analysis of the relationships between the level of physical activity and GMC	Children with higher levels of physical activity presented better motor performance
Lopes et al.^12^	3.616 ♂ and 3.559 ♀ (6 and 14 years)	Analysis of the relationship between BMI and GMC in children	Negative and significant correlation between BMI and GMC
Lopes et al.^39^	315 ♂ and 218 ♀ (9-12 years)	Use of BF% and waist circumference as predictors of GMC level	BF% and waist circumference showed low precision in the prediction of the GMC level
Lopes et al.^36^	3344 ♂ and 3281 ♀ (6-11 years)	Analysis of the relationship between BMI and GMC in children	Children with lower GMC levels have high risks of overweight/obesity
Luz et al.^4^	73 ♂ (8 years)	Analysis of the relationships between maturational state and GMC and mediation by anthropometric variable	Association between physical maturation and GMC performance with mediation of waist circumference
Martins et al.^37^	143 ♂ and 142 ♀ (6-10 years)	Longitudinal analysis of the relations between BMI and GMC	At the end of five years of follow-up, GMC was negatively associated with changes in BMI
Melo et al.^38^	794 ♂/♀ (6-9 years)	Analysis of the associations between the BMI groups (eutrophic, overweight and obese) in MC	MC is moderately and negatively associated with BMI
Moura-dos-Santos et al.^30^	251 ♂ and 232 ♀ (7-10 years)	Analysis of birth weight relationships with GMC	Birth weight does not influence GMC
Vandendriessche et al.^10^	78 ♂ football players (15-16 years)	Analysis of relations of biological maturation with GMC	For young football players, maturation has little influence on GMC
Vandorpe et al.^29^	1.297 ♂ and 1.173 ♀ (6-11 years)	Analysis of the validity of the KTK for children	KTK is a valuable tool for analyzing the GMC of children

♂: boys; ♀: girls; KTK: *Körperkoordinationstest für Kinder*; GMC: gross motor coordination; BMI: body mass index; BF%: body fat percentage; MC: motor coordination.

**Table 4:** Studies (n=6) using the *Körperkoordinationstest für Kinder* together with other motor tests.

1^st^ Author	Sample	Methodological characteristics	Main findings
Bardid et al.^45^	323 ♂ and 315 ♀ (5-6 years)	Comparison between the KTK and the MOT 4-6	Moderate positive associations between tests
Catenassi et al.^46^	27♂ and 11♀ (4-7 years)	Using the KTK and the TGMD-2 to analyze MC and BMI	There was no equivalence in the children’s responses to the tests
Fransen et al.^24^	1.300 ♂ and 1.185 ♀ (6-11 years)	Comparison between the KTK and the BOT-2	Strong positive associations between tests
Lopes et al.^47^	21 ♂/♀ (6 and 7 years)	Using the KTK and the TGMD-2 to analyze MC and fundamental motor skills	The authors did not report comparisons between the test scores
Rudd et al.^14^	86 ♂ and 72 ♀ (6-12 years)	Comparison between the KTK and the TGMD-2 for movement competence analysis	The tests can access discrete aspects of movement competence
Van Aken et al.^43^	38 ♂ and 18 ♀	Comparison between the KTK and the M-ABC for GMC analysis in children with DiGeorge Syndrome	There are positive associations between the scores obtained in the tests with children with DiGeorge Syndrome

♂: boys; ♀: girls; KTK: *Körperkoordinationstest für Kinder*; MOT 4-6: Motor-Proficiency-Test for Children between 4 and 6 Years of Age; TGMD-2: Test of Gross Motor Development 2; MC: motor coordination; BMI: body mass index; M-ABC: Movement Assessment Battery for Children; GMC: gross motor coordination; BOT-2: Bruininks-Oseretsky Test of Motor Proficiency 2.

Tables 2, 3 and 4 highlight the sample size of the studies conducted by Vandorp et al,^29^ Fransen et al.^24^ and Lopes et al., the latter with more than three thousand individuals. Only the study by Laukkanen et al.^9^ is unclear on whether the investigation was conducted with male or female infants.

## DISCUSSION

The objectives of this review were to identify the variables that exert significant influence on the KTK motor test and to analyze other tests that have positive or negative associations with KTK. After analysis of the manuscripts, the main finding of this review was that body composition is the external variable with greater power of influence on the KTK test scores. In addition, the M-ABC test appears to be the one with the highest correlation with KTK when comparing the results of both tests.

Motor performance tends to be influenced by the physical aspects of the individuals evaluated, such as height, body fat percentage, gender and even birth weight.^1^ ^,^ ^30^ ^,^ ^31^ ^,^ ^32^ Studies show that the maturational aspect presents a negative correlation with the motor performance mainly with the backwards gait task, in which children who have a slightly delayed biological maturation show better performances.^14^ ^,^ ^33^ ^,^ ^34^

Freitas et al.^35^, in their research with 429 children aged seven to ten years, identified that maturation had insignificant influence on motor tests, noting that maturational aspects are not directly related to better performance in tasks that assess motor skills.^13^ In this perspective, Vandendriessche et al.^10^ found that the results of motor coordination do not present a positive correlation with aspects related to maturation or physical conditioning.

Seeking to associate the maturational period with motor coordination through the KTK, Rocha et al.^34^ divided 50 girls into two groups according to the presence or absence of menarche and observed that menarche did not influence motor performance.

Body composition is a variable that exerts a direct influence on motor performance regardless of the age and gender of the individual evaluated.^33^ ^,^ ^36^ ^,^ ^37^ ^,^ ^38^ In addition to this concern, care should be taken regarding the health risks caused by overweight. Different methods for measuring body composition in children may be related to motor performance, unlike adults, in which the body mass index (BMI) does not provide fully reliable data, since the amount of muscle mass influences the result. The study by Lopes et al.^39^ found relationships of BMI values, waist circumference, fat percentage and waist/height ratio with motor performance, thus validating all of these methods.

Overweight children aged 10 to 12 years tend to have significantly lower motor performance scores when compared to children aged 5 to 7 years.^15^ The influence of body composition on children’s motor performance seems to be more evident at 11 years of age, but this correlation tends to decrease at 14 years.^12^

With daily physical exercise and body mass reduction, the influence of fat percentage on motor performance tends to decrease, but there is no significant difference in three months of intervention, thus requiring a longer time of practice of physical exercise so that statistically significant changes can be noted.^16^ ^,^ ^33^ ^,^ ^40^ ^,^ ^41^ In the KTK test, only the balance beam task tends to present positive results in a short period of intervention.^8^

Other factors that can positively influence the improvement of children’s motor performance are training with overload^42^ and the socio-affective aspect during the intervention, as demonstrated by Laukkanen et al.^9^ when they compared the motor performance of children who practiced exercise regularly with the encouragement of family members with children who only practiced. The authors observed that the children who performed the intervention with the motivational incentive of the family presented greater gains in motor performance.

Therefore, according to the main findings of the studies listed here, the children’s body composition has a strong influence on the scores obtained in KTK tasks. In addition, this relationship becomes relevant given the limitations that children with motor disorders go through to develop their daily physical practices, in addition to these disorders being possible predictors for overweight and obesity.^12^ As an intervening variable, the regular practice of physical activities has a significant weight in the KTK scores.

Concerning the different motor tests, there is a primary interest in determining the different motor disorders more accurately. To this end, the statistical correlation of the results of different motor test batteries seems to be an efficient strategy. In this perspective, Van Aken et al.^43^ conducted a study with 56 children (±9.6 years), 28 of whom had a deletion syndrome (DiGeorge syndrome), which has a genetic characteristic, and one of its consequences is a deficit in the pattern of behavior, relating to psychiatric disorders. The authors associated the absence of this disorder with the results of the KTK motor test score, M-ABC test and IQ tests. The results showed that the individuals with the syndrome had statistically lower scores in the motor tests when compared to those in the control group who did not have the syndrome, thus suggesting a positive relation between motor test results. On the other hand, such results can be reversed when there is influence of the environmental factors in the motor development of the learner.^3^ ^,^ ^44^

In one study, Fransen et al.^24^ found positive results in the correlation of the motor tasks of the Bruininks-Oseretsky Test of Motor Proficiency 2 (BOT-2) with the motor coordination tests of the KTK when applying a battery of tests with 2,585 children (both sexes) divided into six groups according to age group (6-7; 8-9; 10-11 years). The authors concluded that there was no significant difference between the genders even when the results of the tests were compared between groups. They also observed that age did not influence this correlation. These findings corroborate the results of the research by Bardid et al.,^45^ which associated the scores of KTK and MOT 4-6 in 638 children aged five to six years. The researchers observed that the correlation was stronger in the gross motor activities of MOT 4-6 when compared to the fine motor activities, perhaps due to the nature of the tasks that make up the tests.

In another study, Rudd et al.^14^ compared the results of the KTK and TGMD-2 tests in children aged 6 to 12 years divided into groups according to age group. The authors observed that there is a positive relationship between the test scores at all ages. From this perspective, they suggest that studies are developed to holistically assess motor movement skills in various cultures to measure the influence of the cultural environment on children’s movement patterns. However, different results can be obtained by assessing children aged between four and seven years.^46^ ^,^ ^47^

Debrabant et al.^48^ performed a comparison of the results between the reaction time tests by both standardized and non-standardized visual stimulation and by evaluation of the visual development through the Beery-Buktenica test.^49^ This is accomplished by requesting the child to draw a series of geometric figures with a time limit, with the lateral transposition task of the KTK. The children selected for the sample were aged between five and 12 years and had obtained a value of at least 15 percentile in the M-ABC test, in addition to the latter being an inclusion criterion in the sample. The authors observed that 9- and 10-year-olds had the best reaction times, and the five- and six-year-olds were more dependent on visual stimuli in the reaction time task even when the stimuli were standardized by sound pacing. Another finding was the relationship of reaction time with the lateral transposition task; even though both use distinct parts of the brain, it seems that cognitive abilities influence motor skill acquisition.

Hanewinkel et al.^25^, in a study with 41 children (±8.1 years) with joint hypermobility, performed the M-ABC, the KTK, the 6-minute walk physical test and the manual grip strength test. The authors observed that 78% of the children classified with normal parameters according to the M-ABC corresponded to 22% in the KTK test; 7.4% of the children presented a probability of risk in the M-ABC, but in the KTK this value corresponded to 36.5%; 14.6% in definitive delay according to the M-ABC accounted for 41% according to the KTK. In the association of motor tests with force production values, the relationships were statistically significant only for the KTK test, and the association of force parameters and body composition with the M-ABC was not reliable.^25^

According to D’hondt et al.,^50^ the M-ABC appears to be the test with the highest positive correlation with the KTK. This may be explained by the similarity of test objectives in diagnosing children with movement difficulties, and such a relationship can be classified as a gold standard for the analysis of motor behavior in children.^51^

Thus, it is important to identify other developed and validated motor coordination tests whose results can be positively related to those found in the KTK tests, with the aim of reducing the probability of errors in the diagnosis of individuals with movement difficulties and increasing the reproducibility of the findings.

Finally, this study presented some limitations. Considering the time when the KTK was created,^17^ ^,^ ^18^ surveying the literature since the creation of the test could provide more reliable information about its various applicabilities. In addition, one factor that may influence the test is the (biological and maturational) age of the children. This variable was not raised in this review mainly due to the size of the different studies that comprehended a significant range of ages, with studies with children aged 4 years to studies with teenagers aged up to 16 years.

Still, it is worth mentioning that, although studies with samples composed of subjects with some type of disability were not included in this review because they did not fulfill the proposed eligibility criteria, some studies with this population that applied KTK pre- and post-intervention with physical exercises showed significant improvements in motor performance.^52^ ^,^ ^53^ ^,^ ^54^ ^,^ ^55^

Having said this and associating such data to the findings of the studies included in this review, increasing physical exercise levels seems to have established itself as an important tool for multiprofessional work, integrating the different health areas that work with the pediatric population.

## CONCLUSIONS

Based on the selected studies, it was safely concluded that the variable that exerts the greatest influence on the level of development of motor coordination in the KTK is body composition. Importantly, but to a lesser extent, regular practice of physical activities also exerts influence on the KTK motor test results. Therefore, it is suggested that health professionals working with the pediatric population (physical education teachers, physicians, physiotherapists, nutritionists) encourage the regular practice of exercises, both to improve body composition and to improve the KTK scores, as this test is a predictor of more physically active behaviors in adult life.

Among the motor coordination assessment tests, the results found in the application of the M-ABC test are considered to be the best when associated with the KTK results, thus evidencing the reliability of the application of the two tests for the diagnosis of children with movement difficulties. However, it is worth mentioning that more association studies between motor tests are necessary for more robust assertions about the subject, thus allowing greater comparability among the tests available to health professionals.
